# Editorial: Recent advancements in musculoskeletal regenerative medicine

**DOI:** 10.3389/fcell.2025.1688419

**Published:** 2025-09-22

**Authors:** Csaba Matta, Boopalan Ramasamy, Elizabeth Vinod

**Affiliations:** ^1^ Department of Anatomy, Histology and Embryology, Faculty of Medicine, University of Debrecen, Debrecen, Hungary; ^2^ Department of Orthopaedics, Royal Adelaide Hospital and Adelaide University, Adelaide, SA, Australia; ^3^ Department of Physiology, Christian Medical College, Vellore, India; ^4^ Centre for Stem Cell Research, (A unit of BRIC-InStem, Bengaluru), Christian Medical College, Vellore, India

**Keywords:** musculoskeletal regenerative medicine, mesenchymal stem cells, cartilage and bone repair, single-cell transcriptomics, smart biomaterials and hydrogels, exosome-mediated signaling, inflammation and immunomodulation, translational orthobiologics

## Introduction

Musculoskeletal regenerative medicine continues to evolve rapidly, offering renewed hope for patients with cartilage, bone, and joint disorders that currently lack durable treatments. This Research Topic features ten original contributions that collectively illustrate how the field is moving beyond traditional repair paradigms, integrating cellular engineering, smart biomaterials, immunomodulatory strategies, exosome signaling, and system-level insights. In this editorial, we contextualize these contributions, highlight their translational and clinical implications, and outline priorities for the future in musculoskeletal regeneration.

## Highlights from the Research Topic

Kim et al. harnessed engineered MSCs expressing a doxycycline-inducible IL-1β “sticky-trap,” a membrane-bound decoy receptor. The construct enables localized binding of IL-1β without systemic release, retaining it at joint surfaces. This innovative design allowed local, matrix-retained cytokine modulation while sparing systemic physiology. Intra-articular injection in a murine destabilization of the medial meniscus (DMM) model reduced cartilage degeneration, synovitis, and MMP-13 expression, while preserving type II collagen, illustrating the feasibility of durable, site-specific control of chronic inflammation, a major unmet need in osteoarthritis (OA).

Peng et al. created a single-cell RNA sequencing atlas of cystic lesions in steroid-induced osteonecrosis of the femoral head and identified eight chondrocyte subsets, including homeostatic, fibrocartilaginous, inflammatory, and hypertrophic. A reparative hypertrophic population expressing *CLIC3* showed osteogenic potential transitioning toward bone. Histology confirmed a cartilage-to-bone interface within lesions, outlining a reparative trajectory involving chondrocyte hypertrophy preceding bone formation. This atlas is a blueprint of *in situ* repair processes in necrotic bone and identifies targetable cell populations for future therapy. This approach highlights how mapping cell states at disease interfaces can unveil targets for regeneration without the need for fully exogenous interventions.

Chen et al. explored the dual role of graphene oxide (GO) in skeletal muscle regeneration and its downstream effects on osteoblasts, showing that larger GO particles (>500 nm) enhanced myoblast proliferation and differentiation via PI3K–Akt signaling and NFATc1 upregulation. Also, exosomes from GO-treated myoblasts also stimulate osteoblastogenesis by upregulating osteogenic genes in MC3T3-E1 cells, suggesting muscle–bone crosstalk. GO acts both as a scaffold that supports myogenic maturation and a modulator of paracrine signaling, with potential to coordinate musculoskeletal repair. By positioning muscle regeneration within the broader musculoskeletal unit, this work underscores the systemic perspective required for tissue repair, where local interventions may have cascading effects across connected tissues.

Firoozi et al. compared meniscus-derived matrix scaffolds from healthy versus OA donors. While both supported fibro-chondrocyte viability and matrix deposition, only healthy donor scaffolds improved integration strength. Subtle biochemical or structural alterations in OA tissue may impair repair outcomes, even when decellularization is adequate. This work highlights the significance of donor tissue quality as an often-overlooked determinant of scaffold success, emphasizing the need for standardized screening or synthetic alternatives.

Ilyas Khan and Anderson-Watters demonstrated the ability of BMP9 in promoting cartilage maturation in immature cartilage explants. BMP9 improved collagen alignment, zonal organization, and proteoglycan content, resembling postnatal cartilage. These changes were accompanied by increased stiffness and matrix remodeling features often lacking in engineered constructs. The study supports BMP9 as a tool to accelerate functional maturation of engineered cartilage and addresses a critical translational gap between engineered and native tissues in load-bearing applications where constructs must not only survive implantation but also withstand mechanical load over time.

Santiago et al. conducted a systematic review and meta-analysis comparing platelet-rich plasma (PRP) versus PRP combined with hyaluronic acid (HA) for hip OA. While both treatments improved pain and function in the short term, PRP alone was more effective in pain relief. Since HA addition did not enhance outcomes and was occasionally linked to discomfort, the results question the additive value of HA and the need for protocol harmonization and evidence-based refinement of orthobiologic interventions before broad clinical uptake.

Desando et al. highlight the translational potential of mechanically isolated stromal vascular fraction (mctSVF) in OA. In a preclinical rabbit model, mctSVF promoted osteochondral repair, mitigated synovial inflammation, and preserved meniscal integrity, matching outcomes seen with expanded adipose stromal cells. By retaining native microarchitecture, mctSVF offers a minimally manipulated, clinically relevant approach to cartilage protection and joint regeneration.

Ivanovska et al. reveal that MSCs adapt dynamically to the OA joint environment. In a murine model, intra-articularly delivered MSCs persisted as a subset capable of modulating macrophage-driven inflammation and supporting cartilage repair. Transcriptomic analysis highlighted disease-stage -dependent activation and identified BRINP3 as a potential regulator of MSC function. These findings emphasize the context-specific plasticity of MSCs, offering insight into tailored cell-based interventions for OA.

Ossanna et al. demonstrate that mechanically isolated stromal vascular fraction (HT-SVF) combined with low -molecular-weight hyaluronic acid (ACP) markedly enhances bone regeneration in a murine calvarial defect model. Compared with enzymatically processed SVF, HT-SVF + ACP improved bone matrix maturity, reduced fibrosis, and accelerated healing. These results underscore the translational potential of mechanically processed SVF as a cost-effective, cell-based regenerative strategy for bone and tissue repair.

Finally, Velot et al. provided a perspective acknowledging the contributions of women in stem cell–based OA therapies and underscoring ongoing disparities in recognition and leadership roles. This piece calls for systemic solutions and reminds us that advancing regenerative medicine is not just a technical endeavor, but also a sociocultural one tied to equity and inclusive leadership.

## Conclusion

Collectively, these contributions demonstrate how musculoskeletal regenerative medicine is shifting toward precision interventions featuring targeted modulation of inflammation, the development of smarter scaffolds and biomaterials, and system-level insights for the interconnected biology of musculoskeletal tissues. The collection emphasizes not only structural regeneration but also functional recovery and long-term integration, which are fundamental for successful clinical outcomes. Through cytokine-neutralizing cell therapies, engineered biomaterials, and exosome-mediated communication, these methods bring truly personalized and biologically informed treatments within reach.

Looking ahead, further progress will depend on robust models, standardized assessments, and a clear focus on both personalization and equity, ensuring the benefits of regenerative science extend widely. We thank all contributors for advancing the science and setting a foundation for the next-generation of clinically robust, translationally relevant, and socially conscious regenerative strategies.

